# Supplementary material for: Aroma Characterization of Roasted Meat and Meat Substitutes Using Gas Chromatography–Mass Spectrometry with Simultaneous Selective Detection and a Dedicated Software Tool, AromaMS
Source: Molecules. 2023 May 8;28(9):3973. doi: 10.3390/molecules28093973 (PMC10179901; doi:10.3390/molecules28093973)
Supplement: Supplementary file 1 [file molecules-28-03973-s001.zip › molecules-2317462-supplementary.pdf]

Supplementary data

Table of contents:

Figure S1. Main screen of AromaMS, facilitating the operator to set the parameters for data processing.

Figure S2. Aministrator screen of AromaMS, facilitating the administrator to set the fundamental parameters for data processing.

Table S1. AromaMS data processing results in Non-target mode - SPME-GC-MS/NPD/FPD(S) analysis of roasted meat aroma.

Table S2. AromaMS data processing results in Non-target mode with selective detectors verification - SPME-GC-MS/NPD/FPD(S) analysis of roasted meat aroma.

Table S3. AromaMS data processing results in Non-target mode with selective detectors verification - SPME-GC-MS/NPD/FPD(S) analysis of roasted spoiled meat aroma.

Table S4. AromaMS data processing results in Non-target mode with selective detectors verification - SPME-GC-MS/NPD/PFPD analysis of roasted meat aroma.

Table S5. AromaMS data processing results in Non-target mode with selective detectors verification - SPME-GC-MS/NPD/PFPD analysis of roasted PB-MS1 aroma.

Table S6. AromaMS data processing results in Non-target mode with selective detectors verification - SPME-GC-MS/NPD/PFPD analysis of roasted PB-MS2 aroma.

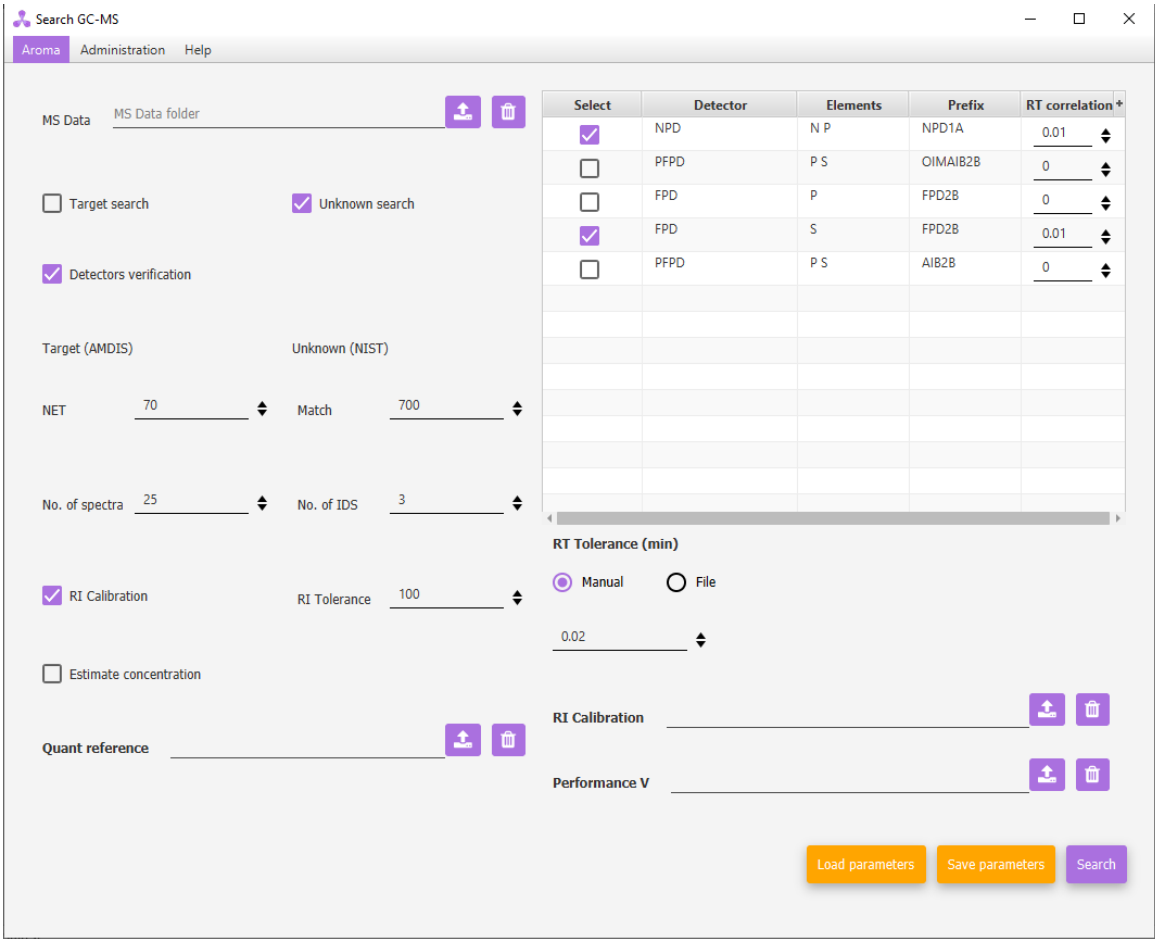

Figure S1. Main screen of AromaMS, facilitating the operator to set the parameters for data processing.

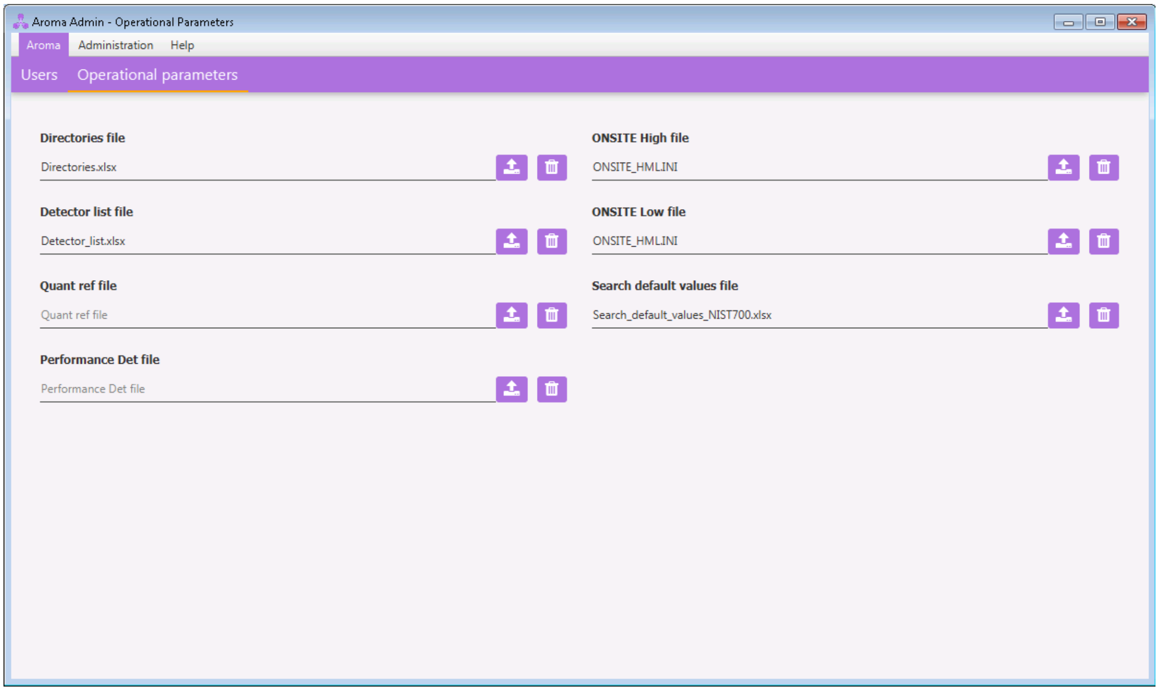

Figure S2. Aministrator screen of AromaMS, facilitating the administrator to set the fundamental parameters for data processing.

Table S1. AromaMS data processing results in Non-target mode - SPME-GC-MS/NPD/FPD(S) analysis of roasted meat aroma.

RT	Name	Formula	CAS	Proba-bility	Match	Area	S/N	RI	NIST RI
0.765	Methylamine, N,N-dimethyl-	C3H9N	75-50-3	82	927	2.4E+08	398	545	502
0.779	Ethanamine, N-methyl-	C3H9N	624-78-2	55	897	2.9E+08	400	547	482
0.913	Oxirane, 2,3-dimethyl-, cis-	C4H8O	1758-33-4	77	801	4.3E+04	13	563	555
1.137	(+)-N-Methylephedrine	C11H17NO	42151-56-4	29	830	3.6E+06	51	591	0
1.166	Propane, 2-methoxy-2-methyl-	C5H12O	1634-04-4	73	930	4.5E+06	101	594	566
1.197	Butane, 2-ethoxy-	C6H14O	2679-87-0	8	729	8.1E+05	54	598	622
1.296	Butanal	C4H8O	123-72-8	36	840	4.0E+06	55	610	593
1.491	Methyl propionate	C4H8O2	554-12-1	96	936	7.5E+05	39	634	627
1.665	Butanal, 3-methyl-	C5H10O	590-86-3	91	918	3.9E+06	73	656	652
1.724	Butanal, 2-methyl-	C5H10O	96-17-3	85	939	2.8E+07	163	663	662
1.738	Disiloxane, hexamethyl-	C6H18OSi2	107-46-0	20	820	6.9E+05	24	665	622
1.738	tert-Butylpentamethyldisiloxane	C9H24OSi2	67875-54-1	51	842	6.9E+05	24	665	0
1.789	Silanediol, dimethyl-	C2H8O2Si	1066-42-8	97	955	7.7E+06	77	671	0
1.811	Aziridine, 1-ethenyl-	C4H7N	5628-99-9	55	875	2.1E+06	42	674	692
1.811	Isoxazole	C3H3NO	288-14-2	89	986	2.1E+06	41	674	590
1.811	(3S)-(-)-3-Acetamidopyrrolidine	C6H12N2O	79286-74-1	25	855	2.1E+06	42	674	0
1.946	Heptane	C7H16	142-82-5	80	879	3.1E+06	71	690	700
1.946	Hexane, 3-methyl-	C7H16	589-34-4	9	801	3.1E+06	71	690	676
2.115	Acetoin	C4H8O2	513-86-0	38	719	1.3E+06	36	711	713
2.159	N,N-Dimethylaminoethanol	C4H11NO	108-01-0	78	967	1.6E+07	121	716	780
2.159	Ethanol, 2-(ethylamino)-	C4H11NO	110-73-6	7	878	1.6E+07	121	716	786
2.184	2,2-Dichloroethyl methyl ether	C3H6Cl2O	34862-07-2	27	729	4.0E+04	12	720	785
2.226	Propanoic acid, 2-hydroxy-, methyl ester, (σ)-	C4H8O3	547-64-8	94	951	1.6E+07	125	725	763
2.226	Ethanol, 2-nitro-	C2H5NO3	625-48-9	3	808	1.6E+07	125	725	658
2.257	2-Hexanone	C6H12O	591-78-6	15	709	1.3E+06	37	729	790
2.271	1H-Pyrrole, 1-methyl-	C5H7N	96-54-8	75	898	2.4E+06	50	730	743
2.343	Pyrrole	C4H5N	109-97-7	82	939	3.2E+06	57	739	755
2.343	3-(2-Propenyl)cyclopentene	C8H12	14564-97-7	5	828	3.2E+06	57	739	801

RT	Name	Formula	CAS	Proba-bility	Match	Area	S/N	RI	NIST RI
2.343	2-Pentyn-4-one	C5H6O	7299-55-0	59	820	3.7E+06	60	739	0
2.354	Pyridine	C5H5N	110-86-1	77	915	4.6E+06	56	741	746
2.359	Disulfide, dimethyl	C2H6S2	624-92-0	98	938	7.4E+06	85	741	746
2.463	Butanoic acid	C4H8O2	107-92-6	97	909	3.8E+06	66	754	802
2.492	Propanenitrile, 3-methoxy-	C4H7NO	110-67-8	82	866	3.8E+04	12	758	750
2.497	2-Propanamine, N-(1-methylethylidene)-	C6H13N	3332-08-9	20	840	8.4E+05	33	758	0
2.497	3-Penten-2-one, 4-amino-	C5H9NO	1118-66-7	39	858	8.4E+05	33	758	0
2.514	2-Butanol, (R)-	C4H10O	14898-79-4	23	900	1.7E+04	10	760	0
2.542	Toluene	C7H8	108-88-3	56	894	1.0E+07	110	764	763
2.542	1,3,5-Cycloheptatriene	C7H8	544-25-2	11	838	1.0E+07	110	764	772
2.583	Thiophene, 2-methyl-	C5H6S	554-14-3	50	888	2.3E+06	51	769	775
2.583	Thiophene, 3-methyl-	C5H6S	616-44-4	36	879	2.3E+06	51	769	786
2.588	2,3-Butanediol, [S-(R*,R*)]-	C4H10O2	19132-06-0	69	899	2.5E+06	78	769	0
2.595	1-Butanol, TMS derivative	C7H18OSi	1825-65-6	40	732	3.7E+06	61	770	798
2.620	Oxetane, 2,4-dimethyl-, trans-	C5H10O	29424-94-0	76	859	2.9E+05	42	773	0
2.664	2,3-Butanediol	C4H10O2	513-85-9	18	863	2.3E+07	182	779	788
2.686	4-Pentyn-2-ol	C5H8O	2117-11-5	31	841	4.5E+06	80	781	0
2.737	L-Lactic acid	C3H6O3	79-33-4	26	968	3.0E+05	33	788	0
2.765	Octane	C8H18	111-65-9	45	839	6.6E+06	88	791	800
2.765	Heptane, 2,4-dimethyl-	C9H20	2213-23-2	29	826	6.6E+06	88	791	821
2.774	Hexanal	C6H12O	66-25-1	89	833	2.5E+06	84	792	801
2.814	Cyclotrisiloxane, hexamethyl-	C6H18O3Si3	541-05-9	89	939	1.6E+07	118	797	851
2.835	Boronic acid, ethyl-	C2H7BO2	4433-63-0	48	848	6.2E+04	19	800	0
2.897	1H-Pyrrole, 1-ethyl-	C6H9N	617-92-5	66	899	5.7E+06	85	807	821
2.929	Pyridine, 2-methyl-	C6H7N	109-06-8	29	912	1.3E+06	62	811	816
2.929	2-Pyridineacetic acid	C7H7NO2	13115-43-0	31	914	1.3E+06	62	811	0
2.931	Pyridine, 4-methyl-	C6H7N	108-89-4	25	901	2.4E+06	60	811	864
2.946	4-Methylthiazole	C4H5NS	693-95-8	42	843	7.8E+05	32	813	818
2.946	Thiazole, 5-methyl-	C4H5NS	3581-89-3	18	804	9.8E+05	35	813	854
3.009	Pyrimidine, 4-methyl-	C5H6N2	3438-46-8	19	901	6.2E+06	96	821	853

RT	Name	Formula	CAS	Proba-bility	Match	Area	S/N	RI	NIST RI
3.011	Pyrazine, methyl-	C5H6N2	109-08-0	68	945	6.9E+06	95	821	829
3.156	1H-Pyrrole, 2-methyl-	C5H7N	636-41-9	72	943	2.2E+06	56	839	850
3.156	1H-Pyrrole, 3-methyl-	C5H7N	616-43-3	26	920	2.2E+06	56	839	858
3.212	3-Furanmethanol	C5H6O2	4412-91-3	30	879	2.1E+06	63	846	835
3.212	2-Furanmethanol	C5H6O2	98-00-0	65	899	2.1E+06	63	846	860
3.224	Acetylacetone	C5H8O2	123-54-6	24	802	1.6E+05	15	848	783
3.258	Ethanol, 2-(2-propenyloxy)-	C5H10O2	111-45-5	5	738	2.1E+06	61	852	769
3.260	2-Hexanone, 5-methyl-	C7H14O	110-12-3	80	851	2.4E+06	58	852	862
3.287	Methoxyacetyl chloride	C3H5ClO2	38870-89-2	85	892	2.4E+04	10	855	0
3.330	Methane, nitroso-	CH3NO	865-40-7	39	940	1.6E+04	10	861	0
3.330	Silane, methyl-	CH6Si	992-94-9	50	947	1.6E+04	10	861	0
3.346	Formic acid, hexyl ester	C7H14O2	629-33-4	9	856	1.0E+06	41	863	914
3.354	Pyridine, 3-methyl-	C6H7N	108-99-6	37	870	2.0E+06	52	864	864
3.363	1H-Pyrrole, 2,5-dimethyl-	C6H9N	625-84-3	21	788	1.3E+06	43	865	902
3.363	Borazine, 1-methyl-	CH8B3N3	21127-94-6	34	800	2.4E+06	58	865	0
3.363	Toluene-D3	C7H5D3	1124-18-1	51	812	2.4E+06	58	865	0
3.398	3-Hexanone, 2-methyl-	C7H14O	7379-12-6	11	790	2.6E+05	20	869	784
3.398	Furan, 2-(dichloromethyl)-tetrahydro-	C5H8Cl2O	931-05-5	22	894	2.2E+05	19	869	0
3.398	Borinic acid, diethyl-, methyl ester	C5H13BO	7397-46-8	33	906	2.2E+05	19	869	0
3.416	2-Pyrazoline, 1,4,5-trimethyl-	C6H12N2	7423-11-2	17	817	4.6E+05	20	871	837
3.416	1H-Pyrazole, 4,5-dihydro-3,5,5-trimethyl-	C6H12N2	3975-85-7	70	854	4.6E+05	20	871	910
3.522	Thiophene, 3,4-dimethyl-	C6H8S	632-15-5	58	754	2.8E+05	24	884	888
3.548	2-Heptanone	C7H14O	110-43-0	90	928	1.4E+07	152	887	891
3.548	2-Hexanone, 4-methyl-	C7H14O	105-42-0	6	826	1.4E+07	152	887	848
3.648	Styrene	C8H8	100-42-5	51	874	1.3E+06	46	900	893
3.648	Bicyclo[4.2.0]octa-1,3,5-triene	C8H8	694-87-1	32	860	1.3E+06	46	900	0
3.670	Heptanal	C7H14O	111-71-7	92	899	7.6E+06	114	902	901
3.670	Hexanal, 3-methyl-	C7H14O	19269-28-4	59	769	6.8E+06	103	902	910
3.711	Pyridine, 2-ethyl-	C7H9N	100-71-0	80	908	9.0E+05	38	907	906

RT	Name	Formula	CAS	Proba-bility	Match	Area	S/N	RI	NIST RI
3.736	2-Cyclopenten-1-one, 2-methyl-	C6H8O	1120-73-6	80	771	1.5E+05	19	911	912
3.747	2(3H)-Furanone	C4H4O2	20825-71-2	6	798	1.9E+07	164	912	914
3.747	2(5H)-Furanone	C4H4O2	497-23-4	91	905	1.9E+07	164	912	920
3.762	1,4-Hexadiene, 3-ethyl-	C8H14	2080-89-9	10	876	8.3E+05	38	914	855
3.776	Butanoic acid, 4-hydroxy-	C4H8O3	591-81-1	95	944	6.9E+06	113	915	933
3.776	Piperazine	C4H10N2	110-85-0	2	777	6.9E+06	113	915	852
3.787	Pyrazine, 2,6-dimethyl-	C6H8N2	108-50-9	45	910	2.0E+07	169	917	917
3.787	Pyrazine, 2,5-dimethyl-	C6H8N2	123-32-0	39	907	2.0E+07	169	917	917
3.809	Methyltartronic acid	C4H6O5	595-98-2	39	967	3.3E+04	11	920	0
3.844	Pyrazine, 2,3-dimethyl-	C6H8N2	5910-89-4	91	865	5.1E+06	86	924	920
4.003	(S)-(+)-2-Pentanol	C5H12O	26184-62-3	29	825	1.6E+04	0	943	0
4.061	Isomaltol	C6H6O3	3420-59-5	23	765	8.2E+05	38	950	989
4.061	1H-Pyrazole, 5-methoxy-1,3-dimethyl-	C6H10N2O	53091-80-8	35	814	3.9E+05	34	950	0
4.068	Pyridine, 2,3-dimethyl-	C7H9N	583-61-9	53	808	2.7E+05	21	951	943
4.068	Pyridine, 2,5-dimethyl-	C7H9N	589-93-5	21	786	2.7E+05	21	951	922
4.103	1H-Pyrrole, 1-butyl-	C8H13N	589-33-3	43	720	1.4E+06	51	956	983
4.115	2-Heptanone, 6-methyl-	C8H16O	928-68-7	95	787	1.1E+06	44	957	956
4.146	Pyridine, 2-(1-methylethyl)-	C8H11N	644-98-4	78	864	6.3E+05	33	961	0
4.165	2-Heptenal, (Z)-	C7H12O	57266-86-1	9	754	9.7E+05	40	963	958
4.165	1-Hexanol, 4-methyl-	C7H16O	818-49-5	8	747	8.0E+05	41	963	953
4.210	Pyridine, 3-ethyl-	C7H9N	536-78-7	34	771	3.2E+06	70	969	961
4.211	Cyclotetrasiloxane, octamethyl-	C8H24O4Si4	556-67-2	65	842	2.1E+07	145	969	994
4.233	1-Heptanol	C7H16O	111-70-6	54	914	2.4E+06	68	972	970
4.233	Formic acid, heptyl ester	C8H16O2	112-23-2	15	885	2.4E+06	68	972	1039
4.250	R-(-)-1,2-propanediol	C3H8O2	4254-14-2	50	918	1.3E+04	0	974	0
4.288	Benzaldehyde	C7H6O	100-52-7	46	877	3.9E+06	79	978	962
4.295	Tetrahydrofurfuryl chloride	C5H9ClO	3003-84-7	71	818	1.6E+05	18	979	930
4.333	1-Octen-3-ol	C8H16O	3391-86-4	81	901	3.5E+06	76	984	980
4.403	Dimethyl trisulfide	C2H6S3	3658-80-8	96	799	2.0E+06	56	993	971
4.425	2-Octanone	C8H16O	111-13-7	76	811	2.5E+06	68	995	991

RT	Name	Formula	CAS	Proba-bility	Match	Area	S/N	RI	NIST RI
4.452	Furan, 2-pentyl-	C9H14O	3777-69-3	82	871	1.7E+06	55	999	993
4.533	Pyrazine, 2-ethyl-5-methyl-	C7H10N2	13360-64-0	32	875	1.5E+07	162	1008	1005
4.533	Pyrazine, 2-ethyl-6-methyl-	C7H10N2	13925-03-6	46	886	1.5E+07	162	1008	1003
4.553	Octanal	C8H16O	124-13-0	89	868	6.3E+06	111	1011	1003
4.566	Pyrazine, trimethyl-	C7H10N2	14667-55-1	94	912	2.0E+07	180	1013	1004
4.628	1-Pentylpyrrolidine	C9H19N	35152-38-6	34	846	1.5E+06	45	1020	1052
4.726	2-Pyrazoline, 1-isobutyl-3-methyl-	C8H16N2	26964-53-4	18	762	1.8E+05	17	1032	1087
4.779	Pyridine, 5-ethyl-2-methyl-	C8H11N	104-90-5	38	900	3.0E+06	61	1039	1031
4.779	4-Isopropylpyridine	C8H11N	696-30-0	47	880	3.1E+06	62	1039	1054
4.779	Pyridine, 3-ethyl-4-methyl-	C8H11N	529-21-5	22	884	3.0E+06	61	1039	1011
4.784	1,4-Cyclohex-2-enedione	C6H6O2	4505-38-8	69	853	5.1E+06	95	1039	1032
4.787	1-Butanamine, 2-methyl-N-(2-methylbutylidene)-	C10H21N	54518-97-7	92	835	1.8E+06	50	1040	1026
4.830	(Z)-1-Phenylpropene	C9H10	766-90-5	26	914	1.2E+05	17	1045	949
4.903	Nitrogen trifluoride	F3N	7783-54-2	32	849	4.6E+05	27	1054	0
4.980	4-Trifluoroacetoxyoctane	C10H17F3O2	116465-17-9	73	805	6.3E+04	13	1064	0
5.011	Butanal, 3,3-dimethyl-2-oxo-, hemihydrate	C6H10O2	77572-68-0	66	863	2.2E+05	20	1067	0
5.024	Thiazole, 4-methyl-2-(1-methylethyl)-	C7H11NS	15679-13-7	83	732	7.8E+05	38	1069	1022
5.061	2-Hydrazinoethanol	C2H8N2O	109-84-2	92	952	2.8E+04	0	1073	0
5.110	p-Cresol	C7H8O	106-44-5	19	797	6.5E+05	33	1079	1077
5.110	1,2-Ethanediol, 1,2-diphenyl-, [R-(R*,R*)]-	C14H14O2	52340-78-0	32	812	6.5E+05	33	1079	0
5.165	Acetophenone	C8H8O	98-86-2	5	716	1.5E+06	50	1086	1066
5.185	2,3-Dimethyl-5-ethylpyrazine	C8H12N2	15707-34-3	11	873	1.9E+07	184	1089	1090
5.185	Pyrazine, 3-ethyl-2,5-dimethyl-	C8H12N2	13360-65-1	68	936	1.9E+07	184	1089	1081
5.239	Pyrazine, 2,6-diethyl-	C8H12N2	13067-27-1	43	788	2.1E+06	58	1095	1084
5.260	Pyrazine, tetramethyl-	C8H12N2	1124-11-4	47	858	4.2E+06	65	1098	1088
5.260	4,4'-Bitriazolyl	C4H4N6	16227-15-9	34	849	4.2E+06	65	1098	0
5.354	3-Pyridinamine, 2-methyl-	C6H8N2	3430-10-2	55	821	2.5E+05	20	1110	0
5.366	Cyclopentane, (1-methylbutyl)-	C10H20	4737-43-3	17	829	4.5E+05	29	1111	1019
5.389	Formamide	CH3NO	75-12-7	62	971	1.3E+05	22	1114	0

RT	Name	Formula	CAS	Proba-bility	Match	Area	S/N	RI	NIST RI
5.393	2-Nonen-1-ol	C9H18O	22104-79-6	5	824	1.1E+07	148	1114	1105
5.393	Nonanal	C9H18O	124-19-6	80	931	1.1E+07	148	1114	1104

Table S2. AromaMS data processing results in Non-target mode with selective detectors verification - SPME-GC-MS/NPD/FPD(S) analysis of roasted meat aroma.

RT	Name	Formula	CAS	Proba-bility	Match	Area	S/N	RI	NIST RI	NPD	FPD
0.765	Methylamine, N,N-dimethyl-	C3H9N	75-50-3	82	927	2.4E+08	398	545	502	V	X
0.779	Ethanamine, N-methyl-	C3H9N	624-78-2	55	897	2.9E+08	400	547	482	V	X
1.811	Aziridine, 1-ethenyl-	C4H7N	5628-99-9	55	875	2.1E+06	42	674	692	V	X
1.811	Isoxazole	C3H3NO	288-14-2	89	986	2.1E+06	41	674	590	V	X
1.811	(3S)-(-)-3-Acetamido pyrrolidine	C6H12N2O	79286-74-1	25	855	2.1E+06	42	674	0	V	X
2.159	N,N-Dimethylaminoethanol	C4H11NO	108-01-0	78	967	1.6E+07	121	716	780	V	X
2.159	Ethanol, 2-(ethylamino)-	C4H11NO	110-73-6	7	878	1.6E+07	121	716	786	V	X
2.271	1H-Pyrrole, 1-methyl-	C5H7N	96-54-8	75	898	2.4E+06	50	730	743	V	X
2.343	Pyrrole	C4H5N	109-97-7	82	939	3.2E+06	57	739	755	V	X
2.354	Pyridine	C5H5N	110-86-1	77	915	4.6E+06	56	741	746	V	X
2.359	Disulfide, dimethyl	C2H6S2	624-92-0	98	938	7.4E+06	85	741	746	X	V
2.492	Propanenitrile, 3-methoxy-	C4H7NO	110-67-8	82	866	3.8E+04	12	758	750	V	X
2.497	2-Propanamine, N-(1-methylethylidene)-	C6H13N	3332-08-9	20	840	8.4E+05	33	758	0	V	X
2.497	3-Penten-2-one, 4-amino-	C5H9NO	1118-66-7	39	858	8.4E+05	33	758	0	V	X
2.583	Thiophene, 2-methyl-	C5H6S	554-14-3	50	888	2.3E+06	51	769	775	X	V
2.583	Thiophene, 3-methyl-	C5H6S	616-44-4	36	879	2.3E+06	51	769	786	X	V
2.897	1H-Pyrrole, 1-ethyl-	C6H9N	617-92-5	66	899	5.7E+06	85	807	821	V	X
2.929	Pyridine, 2-methyl-	C6H7N	109-06-8	29	912	1.3E+06	62	811	816	V	X
2.929	2-Pyridineacetic acid	C7H7NO2	13115-43-0	31	914	1.3E+06	62	811	0	V	X
2.931	Pyridine, 4-methyl-	C6H7N	108-89-4	25	901	2.4E+06	60	811	864	V	X
2.946	4-Methylthiazole	C4H5NS	693-95-8	42	843	7.8E+05	32	813	818	V	X
2.946	Thiazole, 5-methyl-	C4H5NS	3581-89-3	18	804	9.8E+05	35	813	854	V	X
3.009	Pyrimidine, 4-methyl-	C5H6N2	3438-46-8	19	901	6.2E+06	96	821	853	V	X
3.011	Pyrazine, methyl-	C5H6N2	109-08-0	68	945	6.9E+06	95	821	829	V	X
3.156	1H-Pyrrole, 2-methyl-	C5H7N	636-41-9	72	943	2.2E+06	56	839	850	V	X
3.156	1H-Pyrrole, 3-methyl-	C5H7N	616-43-3	26	920	2.2E+06	56	839	858	V	X

RT	Name	Formula	CAS	Proba-bility	Match	Area	S/N	RI	NIST RI	NPD	FPD
3.354	Pyridine, 3-methyl-	C6H7N	108-99-6	37	870	2.0E+06	52	864	864	V	X
3.363	1H-Pyrrole, 2,5-dimethyl-	C6H9N	625-84-3	21	788	1.3E+06	43	865	902	V	X
3.363	Borazine, 1-methyl-	CH8B3N3	21127-94-6	34	800	2.4E+06	58	865	0	V	X
3.510	Methane, nitroso-	CH3NO	865-40-7	34	917	2.3E+04	10	883	0	V	X
3.711	Pyridine, 2-ethyl-	C7H9N	100-71-0	80	908	9.0E+05	38	907	906	V	X
3.776	Piperazine	C4H10N2	110-85-0	2	777	6.9E+06	113	915	852	V	X
3.787	Pyrazine, 2,6-dimethyl-	C6H8N2	108-50-9	45	910	2.0E+07	169	917	917	V	X
3.787	Pyrazine, 2,5-dimethyl-	C6H8N2	123-32-0	39	907	2.0E+07	169	917	917	V	X
3.844	Pyrazine, 2,3-dimethyl-	C6H8N2	5910-89-4	91	865	5.1E+06	86	924	920	V	X
4.061	1H-Pyrazole, 5-methoxy-1,3-dimethyl-	C6H10N2O	53091-80-8	35	814	3.9E+05	34	950	0	V	X
4.068	Pyridine, 2,3-dimethyl-	C7H9N	583-61-9	53	808	2.7E+05	21	951	943	V	X
4.068	Pyridine, 2,5-dimethyl-	C7H9N	589-93-5	21	786	2.7E+05	21	951	922	V	X
4.103	1H-Pyrrole, 1-butyl-	C8H13N	589-33-3	43	720	1.4E+06	51	956	983	V	X
4.103	4(H)-Pyridine, N-acetyl-	C7H9NO	67402-83-9	47	722	1.4E+06	51	956	1038	V	X
4.146	Pyridine, 2-(1-methylethyl)-	C8H11N	644-98-4	78	864	6.3E+05	33	961	0	V	X
4.210	Pyridine, 3-ethyl-	C7H9N	536-78-7	34	771	3.2E+06	70	969	961	V	X
4.403	Dimethyl trisulfide	C2H6S3	3658-80-8	96	799	2.0E+06	56	993	971	X	V
4.533	Pyrazine, 2-ethyl-5-methyl-	C7H10N2	13360-64-0	32	875	1.5E+07	162	1008	1005	V	X
4.533	Pyrazine, 2-ethyl-6-methyl-	C7H10N2	13925-03-6	46	886	1.5E+07	162	1008	1003	V	X
4.566	Pyrazine, trimethyl-	C7H10N2	14667-55-1	94	912	2.0E+07	180	1013	1004	V	X
4.628	1-Pentylpyrrolidine	C9H19N	35152-38-6	34	846	1.5E+06	45	1020	1052	V	X
4.726	2-Pyrazoline, 1-isobutyl-3-methyl-	C8H16N2	26964-53-4	18	762	1.8E+05	17	1032	1087	V	X
4.779	Pyridine, 5-ethyl-2-methyl-	C8H11N	104-90-5	38	900	3.0E+06	61	1039	1031	V	X
4.779	4-Isopropylpyridine	C8H11N	696-30-0	47	880	3.1E+06	62	1039	1054	V	X
4.779	Pyridine, 3-ethyl-4-methyl-	C8H11N	529-21-5	22	884	3.0E+06	61	1039	1011	V	X
4.787	1-Butanamine, 2-methyl-N-(2-methylbutylidene)-	C10H21N	54518-97-7	92	835	1.8E+06	50	1040	1026	V	X
4.903	Nitrogen trifluoride	F3N	7783-54-2	32	849	4.6E+05	27	1054	0	V	X

RT	Name	Formula	CAS	Proba-bility	Match	Area	S/N	RI	NIST RI	NPD	FPD
5.024	Thiazole, 4-methyl-2-(1-methylethyl)-	C7H11NS	15679-13-7	83	732	7.8E+05	38	1069	1022	V	V
5.024	Thiazole, 4-methyl-2-(1-methylethyl)-	C7H11NS	15679-13-7	83	732	7.8E+05	38	1069	1022	V	V
5.061	2-Hydrazinoethanol	C2H8N2O	109-84-2	92	952	2.8E+04	0	1073	0	V	X
5.185	2,3-Dimethyl-5-ethylpyrazine	C8H12N2	15707-34-3	11	873	1.9E+07	184	1089	1090	V	X
5.185	Pyrazine, 3-ethyl-2,5-dimethyl-	C8H12N2	13360-65-1	68	936	1.9E+07	184	1089	1081	V	X
5.239	Pyrazine, 2,6-diethyl-	C8H12N2	13067-27-1	43	788	2.1E+06	58	1095	1084	V	X
5.260	Pyrazine, tetramethyl-	C8H12N2	1124-11-4	47	858	4.2E+06	65	1098	1088	V	X
5.260	4,4'-Bitriazolyl	C4H4N6	16227-15-9	34	849	4.2E+06	65	1098	0	V	X
5.389	Formamide	CH3NO	75-12-7	62	971	1.3E+05	22	1114	0	V	X

Table S3. AromaMS data processing results in Non-target mode with selective detectors verification - SPME-GC-MS/NPD/FPD(S) analysis of roasted spoiled meat aroma.

RT	Name	Formula	CAS	Proba-bility	Match	Area	S/N	RI	NIST RI	NPD	FPD
0.700	Methanethiol	CH4S	74-93-1	97	872	9.4E+05	24	568	473	X	V
1.999	Pyridine	C5H5N	110-86-1	87	906	1.4E+06	31	743	746	V	X
2.021	Disulfide, dimethyl	C2H6S2	624-92-0	97	892	4.7E+06	66	746	746	X	V
3.296	Dimethyl sulfone	C2H6O2S	67-71-0	78	733	4.7E+06	66	908	922	X	V
3.436	1H-Pyrrole, 2-ethyl-	C6H9N	1551-06-0	79	794	8.0E+04	12	925	938	V	X
3.924	2-Butanone, 3,3-dimethyl-1-thiocyanato-	C7H11NOS	57518-71-5	79	840	5.1E+05	29	987	0	V	X
3.948	Dimethyl trisulfide	C2H6S3	3658-80-8	96	876	1.6E+06	48	990	971	X	V
4.273	Thiocyanic acid, 2-propynyl ester	C4H3NS	24309-48-6	76	843	6.1E+04	10	1032	0	V	X
4.828	1,2,4-Triazolo[4,3-b]pyridazin-6(5H)-one	C5H4N4O	20552-63-0	88	870	4.8E+04	0	1102	0	V	X
6.871	Benzene, 2,4-diisocyanato-1-methyl-	C9H6N2O2	584-84-9	77	910	1.7E+07	137	1362	1352	V	X
7.253	2H-Benzimidazol-2-one, 1,3-dihydro-5-methyl-	C8H8N2O	5400-75-9	54	841	5.4E+06	93	1410	0	V	X

Table S4. AromaMS data processing results in Non-target mode with selective detectors verification - SPME-GC-MS/NPD/PFPD analysis of roasted meat aroma.

RT	Name	Formula	CAS	Proba-bility	Match	Area	S/N	RI	NIST RI	NPD	PFPD
0.855	Methylamine, N,N-dimethyl-	C3H9N	75-50-3	79	956	2.4E+07	628	567	502	V	X
1.959	N,N-Dimethylaminoethanol	C4H11NO	108-01-0	45	912	4.5E+05	90	730	780	V	X
1.959	1,2-Ethanediamine, N,N-dimethyl-	C4H12N2	108-00-9	54	914	1.0E+06	83	730	820	V	X
1.995	Pyrazine	C4H4N2	290-37-9	64	817	1.7E+05	48	735	736	V	X
1.995	1,3-Diazine	C4H4N2	289-95-2	33	799	1.7E+05	48	735	736	V	X
2.019	1H-Pyrrole, 1-methyl-	C5H7N	96-54-8	64	827	1.2E+05	39	739	743	V	X
2.019	1,3,5-Triazine	C3H3N3	290-87-9	33	791	7.2E+04	31	739	663	V	X
2.068	Pyridine	C5H5N	110-86-1	87	955	5.1E+05	77	746	746	V	X
2.075	Pyrrole	C4H5N	109-97-7	88	940	6.2E+05	87	747	755	V	X
2.120	Ethanamine, 2-chloro-N,N-dimethyl-	C4H10ClN	107-99-3	38	777	4.3E+04	42	754	772	V	X
2.545	Pyridine, 2-methyl-	C6H7N	109-06-8	32	933	3.0E+05	60	816	816	V	X
2.545	Pyridine, 3-methyl-	C6H7N	108-99-6	20	731	3.6E+04	43	816	864	V	X
2.545	Pyridine, 4-methyl-	C6H7N	108-89-4	21	920	3.0E+05	60	816	864	V	X
2.545	2-Pyridineacetic acid	C7H7NO2	13115-43-0	37	875	3.3E+05	61	816	0	V	X
2.550	4-Methylthiazole	C4H5NS	693-95-8	66	871	8.4E+04	35	817	818	V	X
2.596	Pyrazine, methyl-	C5H6N2	109-08-0	65	944	5.3E+06	261	824	829	V	X
2.649	1H-Pyrrole, 3-methyl-	C5H7N	616-43-3	80	929	3.2E+05	69	831	858	V	X
2.717	1H-Pyrrole, 2-methyl-	C5H7N	636-41-9	81	915	1.4E+05	48	841	850	V	X
2.725	Oxazole, trimethyl-	C6H9NO	20662-84-4	23	790	7.8E+04	29	843	852	V	X
3.219	Pyrazine, 2,5-dimethyl-	C6H8N2	123-32-0	71	934	7.6E+06	294	915	917	V	X
3.219	Pyrazine, 2,6-dimethyl-	C6H8N2	108-50-9	21	908	7.6E+06	294	915	917	V	X
3.251	Phosphorus pentafluoride	F5P	7647-19-0	82	801	6.0E+05	81	920	0	V	X
3.266	Pyrazine, 2,3-dimethyl-	C6H8N2	5910-89-4	98	830	8.6E+05	96	922	920	V	X
3.313	2-Propanone, O-methyloxime	C4H9NO	3376-35-0	94	855	6.5E+03	0	929	0	V	X
3.821	Pyrazine, 2-ethyl-5-methyl-	C7H10N2	13360-64-0	18	868	2.2E+06	157	1004	1005	V	X
3.821	Pyrazine, 2-ethyl-6-methyl-	C7H10N2	13925-03-6	72	903	2.2E+06	157	1004	1003	V	X

RT	Name	Formula	CAS	Proba-bility	Match	Area	S/N	RI	NIST RI	NPD	PFPD
3.853	Pyrazine, trimethyl-	C7H10N2	14667-55-1	79	902	4.5E+06	170	1009	1004	V	X
3.889	1H-Pyrrole-2-carboxaldehyde, 1-methyl-	C6H7NO	1192-58-1	65	794	1.4E+05	37	1014	1016	V	X
3.951	4(H)-Pyridine, N-acetyl-	C7H9NO	67402-83-9	70	757	2.0E+05	41	1023	1038	V	X
3.964	Pyrazine, 2-ethenyl-6-methyl-	C7H8N2	13925-09-2	83	866	1.6E+05	37	1025	1031	V	X
3.964	Pyrazine, 2-ethenyl-5-methyl-	C7H8N2	13925-08-1	16	812	1.6E+05	37	1025	1032	V	X
4.245	Ethanone, 1-(1H-pyrrol-2-yl)-	C6H7NO	1072-83-9	46	886	2.4E+05	44	1066	1063	V	X
4.245	3-Acetyl-1H-pyrroline	C6H7NO	1072-82-8	52	889	2.4E+05	44	1066	0	V	X
4.355	Pyrazine, 2,6-diethyl-	C8H12N2	13067-27-1	12	879	3.5E+06	169	1083	1084	V	X
4.355	Pyrazine, 3-ethyl-2,5-dimethyl-	C8H12N2	13360-65-1	75	945	3.5E+06	169	1083	1081	V	X
4.404	2,5-Pyrrolidinedione, 1-methyl-	C5H7NO2	1121-07-9	33	797	2.2E+05	42	1090	1117	V	X
4.414	2,3-Dimethyl-5-ethylpyrazine	C8H12N2	15707-34-3	33	756	9.6E+05	82	1091	1090	V	X
4.414	Pyrazine, 2-ethyl-3,5-dimethyl-	C8H12N2	13925-07-0	26	751	9.6E+05	82	1091	1084	V	X
4.659	1-(6-Methyl-2-pyrazinyl)-1-ethanone	C7H8N2O	22047-26-3	68	908	3.5E+05	57	1127	1092	V	X
4.659	2-Acetyl-3-methylpyrazine	C7H8N2O	23787-80-6	12	827	2.5E+05	53	1127	1086	V	X
4.659	1-(2-Methylpyrimidin-4-yl)ethan-1-one	C7H8N2O	67860-38-2	11	845	3.5E+05	57	1127	1052	V	X
4.820	5H-5-Methyl-6,7-dihydrocyclopentapyrazine	C8H10N2	23747-48-0	74	731	1.5E+05	35	1151	1144	V	X
4.835	Pyrazine, 2,3-diethyl-5-methyl-	C9H14N2	18138-04-0	81	797	1.6E+05	40	1153	1158	V	X
5.113	1,3-Dimethylimidazolidine-2,4-dione	C5H8N2O2	24039-08-5	82	875	1.9E+05	42	1194	1193	V	X
5.125	Ethanone, 1-(3,5-dimethylpyrazinyl)-	C8H10N2O	54300-08-2	84	755	2.3E+04	14	1196	1174	V	X
5.145	2-Pyridinebutanoic acid	C9H11NO2	0	67	844	4.7E+04	23	1199	0	V	X

RT	Name	Formula	CAS	Proba-bility	Match	Area	S/N	RI	NIST RI	NPD	PFPD
5.166	Pyrazine, 2-methyl-5-(1-propenyl)-, (E)-	C8H10N2	18217-82-8	14	749	1.9E+05	45	1202	1133	V	X
5.166	Pyrazine, 2-methyl-6-(1-propenyl)-, (E)-	C8H10N2	18217-81-7	41	773	1.9E+05	45	1202	1107	V	X
5.311	Pyrazine, 2,5-dimethyl-3-(1-propenyl)-, (Z)-	C9H12N2	55138-73-3	57	814	7.2E+04	33	1223	0	V	X
5.313	Pyrazine, 3,5-dimethyl-2-(1-propenyl)-, (Z)-	C9H12N2	55138-74-4	13	746	8.3E+04	30	1224	1200	V	X
5.455	2-Isoamyl-6-methylpyrazine	C10H16N2	91010-41-2	62	716	3.5E+04	20	1245	1249	V	X
5.455	2,5-Dimethylpyrimidine	C6H8N2	22868-76-4	81	809	9.8E+03	20	1245	0	V	X

Table S5. AromaMS data processing results in Non-target mode with selective detectors verification - SPME-GC-MS/NPD/PFPD analysis of roasted PB-MS1 aroma.

RT	Name	Formula	CAS	Proba-bility	Match	Area	S/N	RI	NIST RI	NPD	PFPD
0.854	Methylamine, N,N-dimethyl-	C3H9N	75-50-3	72	952	1.2E+06	127	567	502	V	X
0.950	3-Pentanamine	C5H13N	616-24-0	24	937	3.3E+05	58	581	0	V	X
0.977	Thiirane	C2H4S	420-12-2	67	963	6.8E+04	24	585	598	X	V
0.977	Ethylenediamine	C2H8N2	107-15-3	40	964	2.8E+04	17	585	612	V	X
1.027	Dimethyl sulfide	C2H6S	75-18-3	54	907	2.2E+04	17	593	520	X	V
1.027	Borane-methyl sulfide complex	C2H9BS	13292-87-0	43	901	2.2E+04	17	593	0	X	V
1.614	3-Aminopyrazole	C3H5N3	1820-80-0	58	833	1.6E+04	13	679	0	V	X
1.944	N,N-Dimethylaminoethanol	C4H11NO	108-01-0	59	940	3.6E+06	170	728	780	V	X
1.991	1,3-Diazine	C4H4N2	289-95-2	12	810	1.7E+05	43	735	736	V	X
1.991	Pyrazine	C4H4N2	290-37-9	85	880	1.7E+05	43	735	736	V	X
2.015	1,3,5-Triazine	C3H3N3	290-87-9	58	876	4.8E+04	26	738	663	V	X
2.062	Pyridine	C5H5N	110-86-1	85	954	1.3E+06	122	745	746	V	X
2.072	Pyrrole	C4H5N	109-97-7	87	962	4.7E+05	79	747	755	V	X

RT	Name	Formula	CAS	Proba-bility	Match	Area	S/N	RI	NIST RI	NPD	PFPD
2.114	Ethanamine, 2-chloro-N,N-dimethyl-	C4H10ClN	107-99-3	71	909	2.8E+05	73	753	772	V	X
2.205	2-Methylpiperidine	C6H13N	109-05-7	38	850	6.0E+05	72	766	793	V	X
2.205	2-Propanamine, N-(1-methylethylidene)-	C6H13N	3332-08-9	41	852	6.0E+05	72	766	0	V	X
2.409	Propane, 2-methyl-2-nitro-	C4H9NO2	594-70-7	28	857	9.8E+05	87	796	0	V	X
2.473	Thiazole, 2-methyl-	C4H5NS	3581-87-1	59	857	3.8E+04	22	806	815	V	X
2.508	1H-Pyrrole, 1-ethyl-	C6H9N	617-92-5	52	811	2.0E+05	47	811	821	V	X
2.543	Pyridine, 4-methyl-	C6H7N	108-89-4	47	860	1.7E+04	34	816	864	V	X
2.543	Pyridine, 3-methyl-	C6H7N	108-99-6	33	849	1.7E+04	34	816	864	V	X
2.595	Pyrazine, methyl-	C5H6N2	109-08-0	65	949	2.8E+06	174	823	829	V	X
2.651	1H-Pyrrole, 2-methyl-	C5H7N	636-41-9	60	896	4.3E+05	61	832	850	V	X
2.716	1H-Pyrrole, 3-methyl-	C5H7N	616-43-3	37	883	1.5E+05	47	841	858	V	X
2.723	Oxazole, trimethyl-	C6H9NO	20662-84-4	37	775	3.7E+04	18	842	852	V	X
2.723	Oxazolidin-2-one, N-propanoyl-	C6H5NO3	143800-42-4	38	861	2.5E+04	15	842	0	V	X
2.723	N-(1H-Imidazol-4-ylmethylidene)hydroxylamine	C4H5N3O	57090-90-1	35	859	2.5E+04	15	842	0	V	X
3.036	Isopropylamine, N-acetyl-	C5H11NO	1118-69-0	23	704	1.1E+05	31	888	883	V	X
3.074	1-Butylpyrrolidine	C8H17N	767-10-2	23	877	1.4E+05	32	894	944	V	X
3.074	1-Butanamine, N-methyl-N-2-propenyl-	C8H17N	24209-62-9	28	866	2.3E+04	32	894	0	V	X
3.074	4-Methyleneproline	C6H9NO2	0	27	881	1.4E+05	32	894	0	V	X
3.152	1,2-Ethanediamine, N,N-dimethyl-	C4H12N2	108-00-9	22	808	2.8E+05	47	906	820	V	X
3.162	Pyridine, 2-ethyl-	C7H9N	100-71-0	75	935	3.6E+06	186	907	906	V	X
3.250	Pyrazine, ethyl-	C6H8N2	13925-00-3	56	870	6.9E+05	78	920	918	V	X
3.265	Pyrazine, 2,3-dimethyl-	C6H8N2	5910-89-4	61	740	1.2E+06	104	922	920	V	X
3.265	2-Methyl-1,3-oxazole-4-carbonitrile	C5H4N2O	89282-09-7	9	777	6.2E+05	83	922	982	V	X
3.565	Pyridine, 3-ethyl-	C7H9N	536-78-7	64	831	7.8E+04	39	966	961	V	X
3.565	Pyridine, 4-ethyl-	C7H9N	536-75-4	17	799	7.8E+04	39	966	958	V	X
3.820	Pyrazine, 2-ethyl-6-methyl-	C7H10N2	13925-03-6	27	834	6.4E+05	81	1004	1003	V	X

RT	Name	Formula	CAS	Proba-bility	Match	Area	S/N	RI	NIST RI	NPD	PFPD
3.820	Pyrazine, 2-ethyl-5-methyl-	C7H10N2	13360-64-0	67	872	5.4E+05	76	1004	1005	V	X
3.858	5-Hydroxy-2-pyrimidinecarbonitrile	C5H3N3O	345642-86-6	35	782	4.6E+05	72	1009	1638	V	X
3.994	Acetylpyrazine	C6H6N2O	22047-25-2	68	816	8.3E+04	28	1030	1023	V	X
4.082	Ethanone, 1-(2-pyridinyl)-	C7H7NO	1122-62-9	91	812	1.1E+05	34	1043	1034	V	X
4.242	Ethanone, 1-(1H-pyrrol-2-yl)-	C6H7NO	1072-83-9	35	855	4.1E+05	52	1066	1063	V	X
4.242	3-Acetyl-1H-pyrroline	C6H7NO	1072-82-8	64	872	4.1E+05	52	1066	0	V	X
4.354	Pyrazine, 3-ethyl-2,5-dimethyl-	C8H12N2	13360-65-1	45	910	3.9E+05	77	1082	1081	V	X
4.354	Pyrazine, 2,6-diethyl-	C8H12N2	13067-27-1	33	901	3.9E+05	77	1082	1084	V	X
4.405	1-Pyrrolidinecarboxaldehyde	C5H9NO	3760-54-1	83	822	3.5E+05	46	1090	1083	V	X
4.414	Propargylamine	C3H5N	2450-71-7	46	824	9.2E+03	13	1091	0	V	X
4.414	Pentanedinitrile	C5H6N2	544-13-8	39	820	9.2E+03	13	1091	0	V	X
4.614	1,5-Dimethyl-2-pyrrolecarbonitrile	C7H8N2	56341-36-7	73	812	1.0E+05	29	1121	0	V	X
4.667	1H-Isoindole, 2,3-dihydro-	C8H9N	496-12-8	46	843	5.5E+04	23	1129	0	V	X
4.818	5H-5-Methyl-6,7-dihydrocyclopentapyrazine	C8H10N2	23747-48-0	76	714	9.1E+04	29	1151	1144	V	X
4.911	Pyridine, 3-butyl-	C9H13N	539-32-2	76	766	7.7E+04	24	1165	1127	V	X
5.144	Pyridine, 2-pentyl-	C10H15N	2294-76-0	56	924	1.7E+06	128	1199	1202	V	X

Table S6. AromaMS data processing results in Non-target mode with selective detectors verification - SPME-GC-MS/NPD/PFPD analysis of roasted PB-MS2 aroma.

RT	Name	Formula	CAS	Proba-bility	Match	Area	S/N	RI	NIST RI	NPD	PFPD
0.854	Methylamine, N,N-dimethyl-	C3H9N	75-50-3	78	978	4.42E+05	71	567	502	V	X
1.986	Acetic acid, nitro-, methyl ester	C3H5NO4	2483-57-0	81	900	8.22E+04	30	734	0	V	X
1.990	Pyrazine	C4H4N2	290-37-9	84	959	4.24E+05	77	734	736	V	X
2.004	Thiazole	C3H3NS	288-47-1	28	879	7.56E+04	31	737	736	V	X
2.004	Isothiazole	C3H3NS	288-16-4	38	888	7.56E+04	31	737	0	V	X

RT	Name	Formula	CAS	Proba-bility	Match	Area	S/N	RI	NIST RI	NPD	PFPD
2.065	Pyridine	C5H5N	110-86-1	85	918	7.51E+05	79	745	746	V	X
2.070	Pyrrole	C4H5N	109-97-7	75	964	1.13E+06	123	746	755	V	X
2.227	Piperidine, 1-methyl-	C6H13N	626-67-5	31	771	8.66E+04	26	769	779	V	X
2.270	Hydrazine, 1-methyl-1-(2-methylpropyl)-	C5H14N2	20240-63-5	28	841	1.75E+04	13	776	756	V	X
2.389	Pyridine-D5-	C5D5N	7291-22-7	20	870	4.42E+04	24	793	0	V	X
2.477	Thiazole, 2-methyl-	C4H5NS	3581-87-1	54	854	2.63E+04	16	806	815	V	X
2.507	1H-Pyrrole, 1-ethyl-	C6H9N	617-92-5	89	890	1.02E+05	40	811	821	V	X
2.544	Pyridine, 2-methyl-	C6H7N	109-06-8	39	861	1.68E+05	44	816	816	V	X
2.544	Pyridine, 4-methyl-	C6H7N	108-89-4	20	806	1.24E+05	38	816	864	V	X
2.548	4-Methylthiazole	C4H5NS	693-95-8	58	878	5.03E+04	27	817	818	V	X
2.548	Thiazole, 5-methyl-	C4H5NS	3581-89-3	8	746	5.72E+04	28	817	854	V	X
2.548	Isothiazole, 4-methyl-	C4H5NS	693-90-3	71	824	5.72E+04	28	817	0	V	X
2.593	Pyrazine, methyl-	C5H6N2	109-08-0	71	957	8.06E+06	315	823	829	V	X
2.716	1H-Pyrrole, 2-methyl-	C5H7N	636-41-9	74	908	1.50E+05	46	841	850	V	X
2.716	1H-Pyrrole, 3-methyl-	C5H7N	616-43-3	25	884	1.50E+05	46	841	858	V	X
2.873	2-Aminocyanoacetamide	C3H5N3O	6719-21-7	68	893	3.55E+04	20	864	0	V	X
2.881	Pyridine, 3-methyl-	C6H7N	108-99-6	57	749	1.86E+05	43	866	864	V	X
3.037	Isopropylamine, N-acetyl-	C5H11NO	1118-69-0	13	720	2.77E+04	19	889	883	V	X
3.037	Formamide, N-(1,1-dimethylethyl)-	C5H11NO	2425-74-3	61	765	2.77E+04	19	889	915	V	X
3.163	Pyridine, 2-ethyl-	C7H9N	100-71-0	48	767	7.84E+05	96	907	906	V	X
3.218	Pyrazine, 2,5-dimethyl-	C6H8N2	123-32-0	56	939	3.92E+06	215	915	917	V	X
3.263	Pyrazine, ethyl-	C6H8N2	13925-00-3	26	815	2.33E+06	133	922	918	V	X
3.263	1H,4H,5H,6H-Cyclopenta[c]pyrazole	C6H8N2	15409-55-9	25	814	2.33E+06	133	922	0	V	X
3.370	Pyrazine, ethenyl-	C6H6N2	4177-16-6	89	829	1.09E+05	33	938	946	V	X
3.564	Pyridine, 3-ethyl-	C7H9N	536-78-7	72	812	2.96E+05	53	966	961	V	X
3.819	Pyrazine, 2-ethyl-6-methyl-	C7H10N2	13925-03-6	58	883	1.28E+06	122	1004	1003	V	X
3.819	Pyrazine, 2-ethyl-5-methyl-	C7H10N2	13360-64-0	15	850	1.28E+06	122	1004	1005	V	X
3.853	Pyrazine, trimethyl-	C7H10N2	14667-55-1	89	881	1.22E+06	105	1009	1004	V	X

RT	Name	Formula	CAS	Proba-bility	Match	Area	S/N	RI	NIST RI	NPD	PFPD
3.879	1H-Pyrrole-2-carboxaldehyde	C5H5NO	1003-29-8	97	849	5.41E+05	77	1013	1012	V	X
3.888	2-Methyl-1,3-oxazole-4-carbonitrile	C5H4N2O	89282-09-7	31	874	6.42E+04	25	1014	982	V	X
3.963	Pyrazine, 2-ethenyl-6-methyl-	C7H8N2	13925-09-2	78	722	1.99E+05	38	1025	1031	V	X
3.963	1,2,4-Triazolo[4,3-b]pyridazine	C5H4N4	274-83-9	45	831	8.67E+04	25	1025	0	V	X
3.963	1H-Imidazo(4,5-d)pyridazine	C5H4N4	273-00-7	35	825	8.67E+04	25	1025	0	V	X
3.992	Acetylpyrazine	C6H6N2O	22047-25-2	73	922	2.35E+05	56	1029	1023	V	X
4.201	Phenol, 4-amino-2-methyl-	C7H9NO	2835-96-3	42	875	3.39E+04	21	1060	0	V	X
4.241	Ethanone, 1-(1H-pyrrol-2-yl)-	C6H7NO	1072-83-9	60	889	4.26E+05	63	1066	1063	V	X
4.298	4-Amino-6-hydroxypyrimidine	C4H5N3O	1193-22-2	55	932	4.62E+04	18	1074	0	V	X
4.353	Pyrazine, 3-ethyl-2,5-dimethyl-	C8H12N2	13360-65-1	62	917	8.11E+05	83	1082	1081	V	X
4.402	2,5-Pyrrolidinedione, 1-methyl-	C5H7NO2	1121-07-9	39	717	3.45E+04	20	1090	1117	V	X
4.415	2,3-Dimethyl-5-ethylpyrazine	C8H12N2	15707-34-3	46	700	2.53E+05	48	1092	1090	V	X
4.606	1-(6-Methyl-2-pyrazinyl)-1-ethanone	C7H8N2O	22047-26-3	62	817	1.49E+05	39	1120	1092	V	X
4.613	Pyrazine, (1-methylethenyl)-	C7H8N2	38713-41-6	40	840	2.54E+05	49	1121	1059	V	X
4.613	Pyrazine, isopropenyl-	C7H8N2	34413-32-6	77	875	1.83E+05	43	1121	0	V	X
4.613	1,5-Dimethyl-2-pyrrolecarbonitrile	C7H8N2	56341-36-7	42	841	2.54E+05	49	1121	0	V	X
4.657	2-Acetyl-3-methylpyrazine	C7H8N2O	23787-80-6	43	798	1.50E+05	40	1127	1086	V	X
4.863	Pyrazine, 3,5-diethyl-2-methyl-	C9H14N2	18138-05-1	27	805	7.41E+04	28	1157	1162	V	X
4.863	2,3,5-Trimethyl-6-ethylpyrazine	C9H14N2	17398-16-2	16	790	7.41E+04	28	1157	1163	V	X
